# Cancer: A Study of Three Hundred and Ninety-seven Cases of Cancer of the Female Breast, with Clinical Observations

**Published:** 1887-05

**Authors:** 


Cancer: A Study of three Hundred and Ninety-seven Cases of Cancer of the Female Breast, with Clinical Observations. *By* Willard Parker, M.D., *New York and London:* G. P. Putnam's Sons.

Every one familiar with the life of Dr. Parker knows the shrewd practical sense which gave him his large and successful practice. This brochure reflects this quality as much in what it avoids as in what it attempts.

The record extends over fifty years of practice. The author has tabulated nearly four hundred cases of "cancer of the female breast," under various headings among which are "family history," "manifestation of disease before and after menopause," "age at commencement," "age at operation," "number of operations," "lived after operation," "variety of cancer," etc. The table is introduced by a commentary of about sixty pages.

His diagnoses were, probably, purely clinical. The names given to the growths indicate this. Indeed, it is only within the last few years covered by the record that there was any other method of diagnosis in such cases. But even with care and skill the clinical method is liable to error.

Dr. Parker holds the causes of the disease to be, First, "too much nitrogenous food and too little exercise;" Second, "local irritation of some epithelial surface;" Third, "mental affliction;" Fourth, "dysmenorrhœa." He thinks that the tendency to the disease is the result of the habits of the individual; that heredity has little to do with it; and he makes some calculations to show that even the highest proportion of cases given by the advocates of heredity, in which a family taint can be found, is no more than might be expected from accidental coincidence. It is surprising that, although there has been so much discussion as to the heredity of cancerous disease, no one has attempted to trace the history of the descendents of such patients. A case in point occurs to me. The breast of a lady was amputated for "cancer," the disease re-appeared in the axilla and caused her death. This was some eighty or ninety years ago. The history of the growth, which has been many times given to me by eye-witnesses, was exactly that of carcinoma. The patient had many descendents, but no malignant growth has appeared in any of them down to the fifth generation. In a more recent case no malignant growth has appeared down to the third generation; but phthisis developed in the second generation. It does not seem difficult to find many instances among old families, where the history of the descendents of such cases could be traced for generations. If a number of these were carefully compiled and compared with the histories of healthy people, it would go far towards settling the question.

The author does not state how he operated, but unless he differed from his fellows, his earlier methods, at least, were less thorough than those now in use.

He has estimated the duration of the disease in 178 cases; in 100 of these the tumor was removed by the knife. Of these last "there are sixteen cases of long duration * * * which were either living in 1883 or were well when last heard from. Several cases are also living in which the tumors have been removed within the last seven years."

These results are superior to the best European statistics, which are, on the whole, unfavorable. They are better even than those of Gross (*vide* "Tumors of the Mammary Gland"). The question of diagnosis at once arises here. The author insists on the necessity of attention to hygiene in treating the disease. It is not sufficient simply to remove the growth, "we must change the diathesis." * * "Avoid the so-called predisposing causes,—*i. e.;* unnecessary luxury in mode of life; especially abstain from eating food rich in nitrogen. Urge your patient to take sufficient exerci·e; point out to her the necessity of cleanliness, and of avoiding all articles of dress that would induce irritation of the skin by pressure. Persuade her o cultivate cheerfulness of disposition, and regulate her various functions, particularly that of menstruation. All this will tend to forestall the development of the disease."

There is nothing new in the book and little that can be turned to par- cular use, but, it is interesting as a record of the views and experiences of one of the older school of practitioners.

